# Early Biological Reaction to a Synthetic Hybrid Patch Inducing Tissue Ingrowth as a Senning Atrial Baffle

**DOI:** 10.1016/j.atssr.2026.02.030

**Published:** 2026-03-11

**Authors:** Akira Yamaguchi, Hiroomi Murayama, Shintaro Nemoto

**Affiliations:** 1 Department of Cardiovascular Surgery, Kids’ Heart Center, Aichi Children’s Health and Medical Center, Aichi, Japan; 2 Department of Thoracic and Cardiovascular Surgery, Osaka Medical College, Osaka, Japan

## Abstract

We report a case in which a new synthetic hybrid patch inducing autologous tissue ingrowth was used to replace the obstructed Senning atrial baffle in a double switch operation for congenitally corrected transposition of the great arteries. However, recurrent baffle obstruction developed after 10 months postoperatively and was removed. Histology of the excised specimen showed promising tissue ingrowth but cautionary peripheral folding of the patch in this clinically challenging condition.

The Videos can be viewed in the online version of this article [https://doi.org/10.1016/j.atssr.2026.02.030] on https://www.annalsthoracicsurgeryshortrep.org

The double switch operation for congenitally corrected transposition of the great arteries may require the use of a baffle during the Senning procedure because of its unique anatomical configuration.^1^ However, reoperation due to baffle obstruction occasionally occurs. A new synthetic hybrid fabric (SHF) inducing tissue ingrowth^2^^,^^3^ was used as a replacement of the obstructed previous baffle; however, it developed recurrent obstruction 10 months later. We present the histologic findings of the excised SHF, demonstrating its in vivo behavior when used as a Senning baffle.

A 3.5-year-old boy with congenitally corrected transposition of the great arteries was diagnosed with symptomatic pulmonary congestion and pleural effusion at 6 months after the double switch operation consisting of arterial switch operation and Senning procedure. The obstructed Senning baffle of a 0.6-mm expanded polytetrafluoroethylene patch (ePTFE, WL Gore & Associates) was replaced. At the redo surgery, the ePTFE patch entirely covered with severely thick fibrous tissue was replaced with a SHF patch (Synfolium, Teijin Medical Technology Co Ltd) (Figures 1A-1C; Supplemental Video 1). The patient's postoperative course was uneventful, and he was discharged 13 days after surgery with wide opening of the route form the pulmonary venous chamber to the mitral valve (Figure 1D).

**Figure 1:**
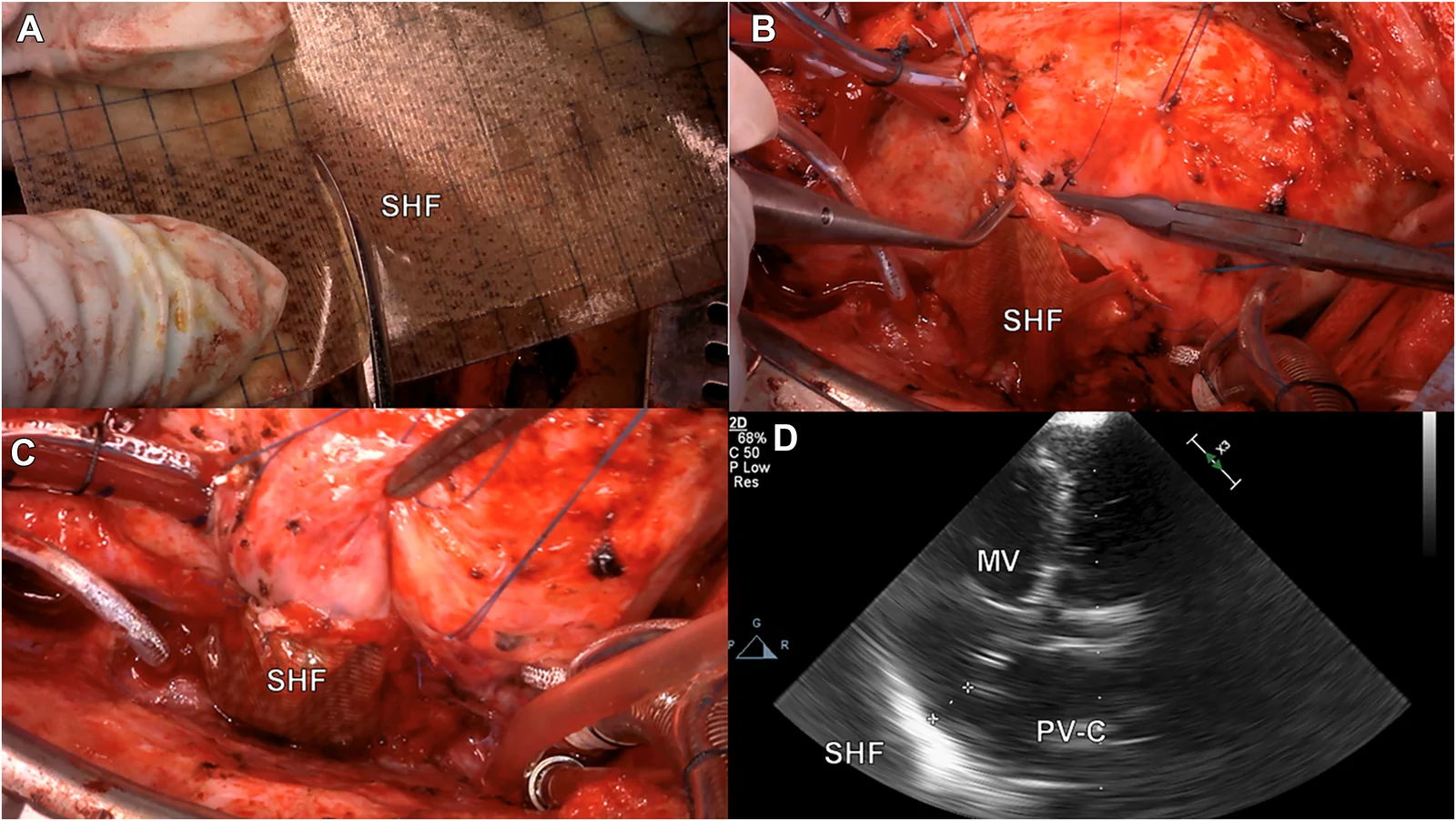
Synthetic hybrid fabric (SHF) implantation as a Senning atrial baffle. (A) Trimming of SHF. (B) Reconstruction of the route from the pulmonary vein (PV) chamber to the mitral valve (MV) using a SHF patch. (C) Installation of a new SHF Senning baffle. (D) Representative echocardiogram at discharge showing wide opening of the route form PV to MV. (PV-C, pulmonary venous chamber.)

However, after 10 months, the peak velocity across the SHF baffle gradually increased to 2.6 m/s with tricuspid regurgitation velocity at 4.6 m/s, with the patch showing obstruction on enhanced computed tomography (Figure 2A). Therefore, a repeat Senning baffle replacement was indicated. The failed SHF patch was removed (Figures 2B, 2C), and the pulmonary venous chamber was reconstructed using the lateral pericardium according to sutureless techniques (Supplemental Video 2). The excised patch was severely thickened with fibrous tissue near the surrounding suture line, except the center (Figure 2D). The patient was discharged hospital 12 days after surgery with an uneventful clinical course.

**Figure 2:**
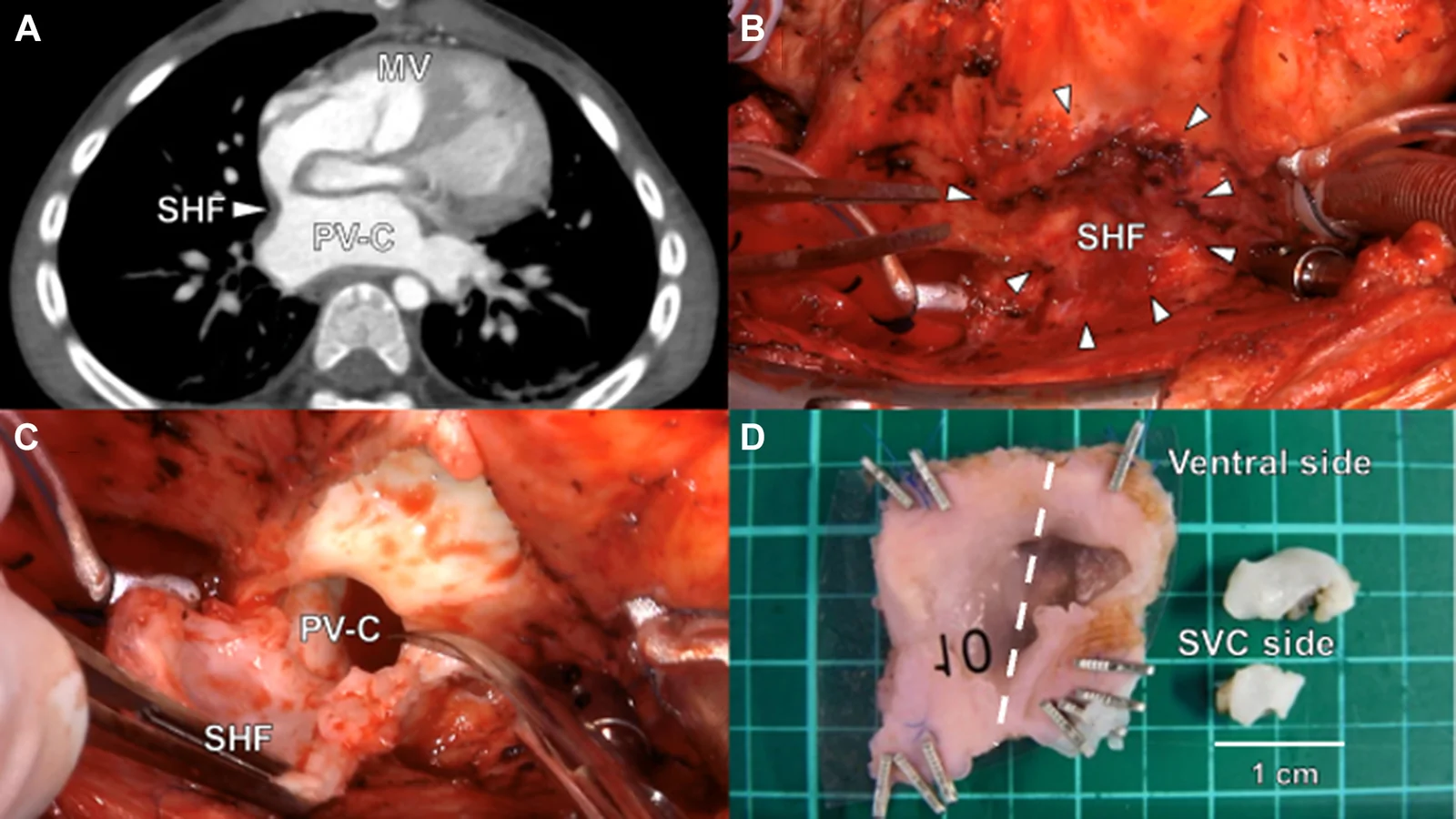
Recurrent obstruction between the pulmonary venous chamber and mitral valve across the Senning baffle. (A) Enhanced computed tomography showing the obstruction across the Synthetic hybrid fabric (SHF) baffle. (B) Appearance of the shrunken SHF. (C) Excision of the thickened and shrunk SHF patch. (D) The luminal side of the excised SHF baffle fixed in formalin. The dotted line indicates the incision line for histologic assessment shown in Figure 3. (MV, mitral valve; PV-C, pulmonary venous chamber; SVC, superior vena cava.)

Histology showed that autologous tissue mainly composed of collagen fibers developed bridging the gelatin degraded portion of the SHF patch (Figures 3A, 3B), and the luminal surface was covered with an endothelial layer (Figure 3C) without calcium deposition (Figure 3D). Notably, the patch was accordion-like in shape within the thick fibrous collagen tissue near the surrounding suture line with a remnant of gelatin and modest macrophage infiltration (Figures 3A, 3B).

**Figure 3:**
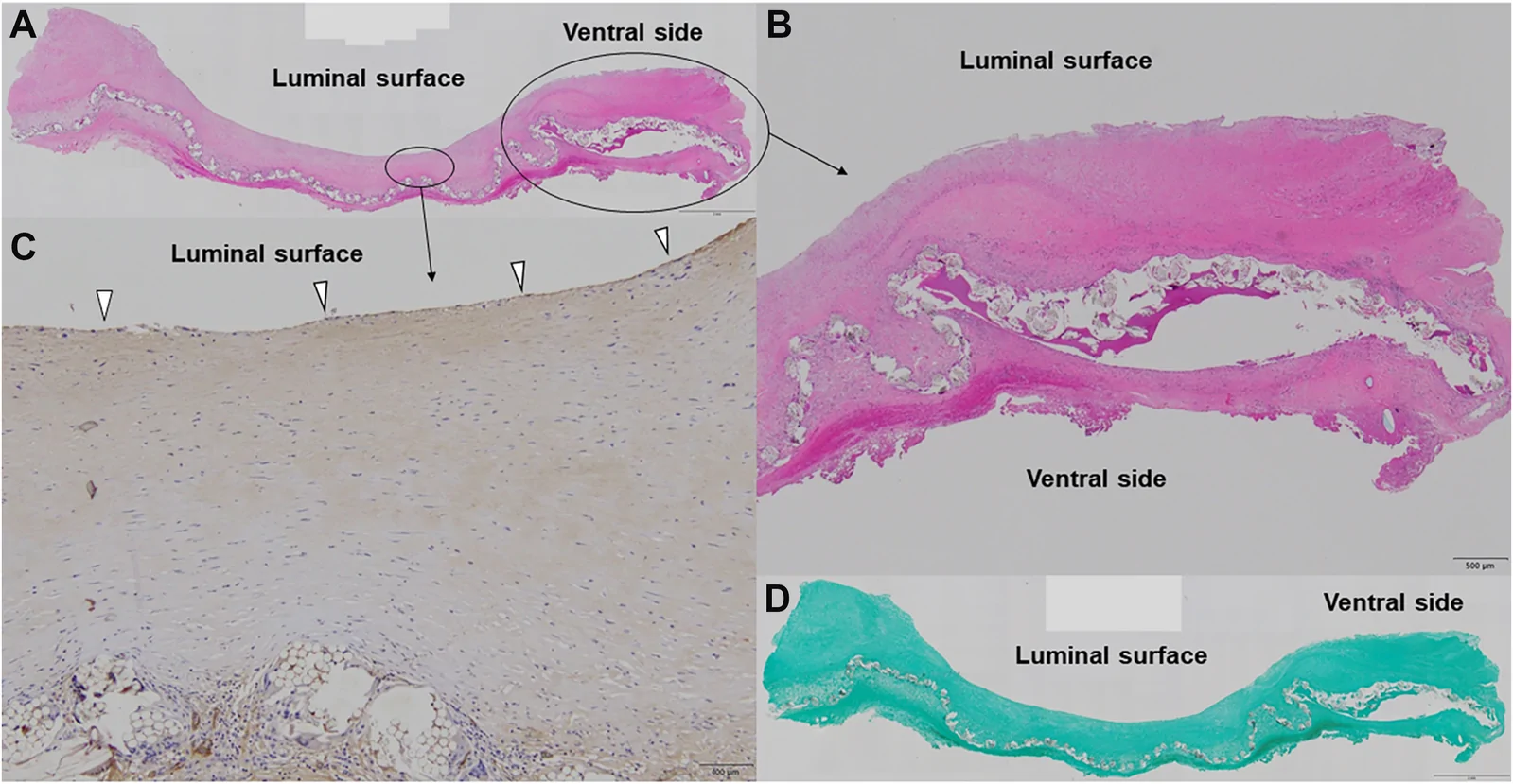
Histologic findings of the vertically incised caudal side of the excised synthetic hybrid fabric (SHF) patch. (A) Hematoxylin and eosin staining of the entire SHF patch at lower magnification (loupe level). (B) Hematoxylin and eosin stain for detailed observation of the thickening and shrinkage of the SHF patch at the surrounding suture line at hyper magnification (x40). (C) The endothelial layer on the luminal surface was detected using von Willbrand staining at hyper magnification (x 100). Arrowheads indicate the endothelial layer. (D) Alizarin red staining to detect calcification in the excised patch (loupe level).

## Comment

The SHF patch is a crosslinked gelatin-coated warp-knitted fabric made from 2 types of yarn: biodegradable poly-L-lactic acid and nonbiodegradable polyethylene terephthalate.^2^^,^^3^ Preclinical studies have shown that the SHF can be successfully replaced with autologous tissue that matures after degradation of gelatin and poly-L-lactic acid with expandability without chronic inflammatory reaction or calcification in canine aortic wall.^2^^,^^3^ The SHF was launched in Japan in 2024 after a successful clinical trial^4^ and is currently undergoing postmarketing surveillance. However, the behavior of SHF in humans has never been histologically examined using excised specimens.

Similar to the findings in the preclinical studies,^2^^,^^3^ typical early signs of autologous tissue ingrowth after gelatin degradation in the SHF were observed throughout the excised Senning baffle specimen as described above. Although the autologous tissue formed on both sides of the baffle, the core fabric itself was folded at the surrounding suture line, possibly resulting in patch thickening and shrinkage, which was different from the ePTFE patch thickening caused by overall pseudointimal proliferation and material deterioration due to foreign body reaction.^5^ This folding shrinkage may have been caused by the contractile force of collagen fibers extending from the surrounding native tissue. Considering that this patch folding did not develop in the canine aorta in preclinical studies and right ventricular outflow to the pulmonary artery in clinical trial, this contractile force may be offset by wall stress generated by intravascular pressure. This folding might have been enhanced by reactive intimal hyperplasia with fibroblast proliferation in children with pulmonary vein stenosis.^6^

Taken together, although the SHF inducing tissue ingrowth could be a promising material in pediatric congenital heart surgery, caution should be exercised when using in low-pressure systems such as a Senning atrial baffle.
